# Exercise Training Interventions as Therapeutic Approaches in Substance Use Disorder Treatment: A Scoping Review of Direct Clinical Outcomes and Implementation Strategies

**DOI:** 10.3390/diseases14080272

**Published:** 2026-07-27

**Authors:** Karim Chamari, Wissem Dhahbi, Sumaia AlMadhoun, James England, Mohammad Mansi, Hala Al Ali, Jallal Toufiq, Khalifa Alkuwari

**Affiliations:** 1 Naufar Centre, Doha P.O. Box 93097, Qatar; karim.chamari@naufar.com (K.C.); sumaya.elmadhoun@naufar.com (S.A.); james.england@naufar.com (J.E.); mohammad.mansi@naufar.com (M.M.); jallal.toufiq@naufar.com (J.T.); khalifa.alkuwari@naufar.com (K.A.); 2 Research Unit “Sport Sciences, Health and Movement”, High Institute of Sports and Physical Education of Kef, University of Jendouba, Kef 8100, Tunisia; 3 Training Department, Police College, Qatar Police Academy, Doha P.O. Box 7157, Qatar

**Keywords:** exercise interventions, aerobic conditioning, resistance training, addiction treatment, relapse prevention, treatment adherence, neuroplasticity, implementation science, precision medicine

## Abstract

Background/Objectives: Substance use disorders (SUDs) remain a major public health challenge, with high relapse rates despite established pharmacological and behavioral treatments. This scoping review maps evidence on structured exercise interventions as therapeutic approaches in SUD treatment, focusing on direct clinical outcomes, substance-specific effects, and implementation strategies. Methods: Following PRISMA-ScR and JBI guidance, searches of PubMed, Scopus, PsycINFO, and SPORTDiscus identified human studies published from 2000 to 31 December 2024 that evaluated supervised exercise interventions in adults with SUDs. Results: Evidence from randomized trials and recent network meta-analyses indicates clinically relevant benefits for abstinence, craving reduction, mood symptoms, cognitive function, treatment retention, and withdrawal management. Moderate-to-vigorous aerobic exercise delivered three to four times weekly for at least 12 weeks, at approximately 180 MET-minutes per week, appears particularly effective, while combined aerobic and muscle-performance protocols may yield broader benefits. Conclusions: Exercise interventions show strong potential as adjuncts to SUD care. Implementation should incorporate individualized prescription, substance-specific safety screening, cardiovascular and psychiatric monitoring, and multidisciplinary coordination. Further long-term randomized trials, mechanistic studies, and cost-effectiveness evaluations are needed.

## 1. Introduction

Substance use disorders (SUDs) constitute a complex neurobiological condition affecting approximately 270 million individuals worldwide, with devastating consequences for individual health, family systems, and societal resources [1]. Despite significant advances in evidence-based treatments, including medication-assisted therapy, cognitive-behavioral interventions, and contingency management, relapse rates consistently exceed 60% within the first year following treatment, indicating substantial limitations in current therapeutic approaches [2]. The resulting economic burden exceeds $440 billion annually in the United States alone, encompassing healthcare costs, criminal justice expenses, and loss of productivity [3]. Addiction involves dysregulation across dopaminergic reward pathways, stress-response mechanisms, and executive function networks, while traditional pharmacological approaches provide incomplete symptom resolution and carry risks of adverse effects [4,5]. Recent network meta-analyses indicate that “standard therapy and exercise” is superior to “standard therapy alone” across addiction domains, with dose–response relationship studies suggesting optimal prescription parameters [6,7].

Exercise interventions in patients with SUD demonstrate therapeutic potential through direct effects on addiction outcomes and recovery trajectories, operating through converging neurobiological, psychological, and physiological mechanisms that target addiction pathophysiology [3]. Evidence suggests that aerobic exercise upregulates BDNF through the PGC-1α/FNDC5 pathway, a mechanism established primarily in preclinical models and proposed to promote synaptic plasticity in regions implicated in addiction. Consistent with this hypothesis, 12-week moderate-to-vigorous exercise protocols have been associated with a BDNF increase averaging 34.7% [8,9]. The dopaminergic reward system is a proposed primary therapeutic target; preclinical and neuroimaging evidence suggests that exercise may normalize D2-receptor availability and enhance dopamine synthesis capacity through cross-stressor adaptation, thereby activating overlapping reward pathways while serving as a competing reinforcer [10].

Exercise interventions may address stress-induced relapse vulnerability partly through modulation of hypothalamic–pituitary–adrenal (HPA) axis reactivity. Exercise (i) modulates cortisol and ACTH dynamics that are frequently altered in addiction, while (ii) balancing autonomic nervous system function [11,12]. Exercise-associated prefrontal cortex neuroplasticity has been linked to partial restoration of gray matter volume and white matter integrity in networks relevant to cognitive control, though causal directionality should incorporate confirmation from longitudinal trials [13,14]. Exercise-induced endogenous opioid and endocannabinoid release has been proposed as an additional mechanism; these neurochemical shifts may attenuate craving and partially offset withdrawal-related neuroinflammation, though direct replacement-therapy equivalence remains to be established [15,16]. High-intensity interval training (HIIT) and anaerobic exercise modalities have emerged as a particularly promising research direction, with preliminary data suggesting enhanced cognitive recovery and accelerated neuroplasticity compared to moderate-intensity protocols. HIIT protocols utilizing 4 × 4 min intervals at 90–95% of maximum heart rate have been associated with substantial effects on executive function restoration and craving reduction in methamphetamine and opioid use disorder patients in a completed trial [17], while two frequently cited HIIT-cognition sources in this review remain at the protocol stage without yet-reported primary outcomes [18,19]. These high-intensity approaches are hypothesized to optimize therapeutic outcomes through rapid BDNF pathway activation and dopaminergic system modulation, though head-to-head mechanistic trials remain limited.

From a psychological perspective, exercise profoundly enhances self-efficacy through mastery experiences and the progressive achievement of goals, which directly correlate with increased confidence in maintaining abstinence and effective treatment engagement [20]. Physiologically, muscle performance training supports endocrine and autonomic regulation by attenuating stress-axis dysregulation, improving cortisol dynamics, autonomic balance, and inflammatory markers, and enhancing metabolic health while counteracting deconditioning commonly observed in chronic substance users [21].

Although recent meta-analyses establish exercise efficacy and dose-response relationships [6,7], our scoping review addresses the implementation gap by systematically mapping evidence on exercise interventions as therapeutic approaches in SUD treatment, examining direct clinical outcomes, substance-specific effects, and implementation strategies for clinical practice. Our review targets healthcare providers, exercise physiologists, and addiction medicine specialists seeking evidence-based guidance for implementing exercise interventions within comprehensive addiction treatment programs.

The specific aims encompass three critical domains, synthesizing evidence for aerobic conditioning, muscle performance training (previously called strength training; we consider that strength is limiting the scope of this type of training that could also encompass, e.g., power and functional capacity), and combined exercise approaches on direct SUD outcomes including (i) substance use reduction, craving intensity, relapse rates, and treatment adherence; (ii) examining substance-specific intervention effects and implementation considerations across alcohol use disorder, opioid use disorder, stimulant use disorders, and cannabis use disorder patients (acknowledging the varied physiological and psychological profiles inherent across different SUDs); and (iii) delivering actionable, mechanism-based clinical recommendations for immediate integration into standard substance use disorder care protocols by identifying optimal prescription parameters, safety protocols, and healthcare system integration strategies for immediate clinical implementation. To maintain a coherent thread, our review is organized across three domains: neurobiological mechanisms, direct clinical outcomes, and implementation strategies. Subsequent sections synthesize evidence by exercise modality and conclude with clinically actionable prescription parameters and deployment considerations.

## 2. Methods

### 2.1. Protocol and Registration

A detailed review protocol was developed a priori, adhering to PRISMA-ScR statement guidelines and prospectively registered with the Open Science Framework (registration number: doi.org/10.17605/OSF.IO/K3E8Z), ensuring transparency, reproducibility, and public access [22,23]. Registration encompassed a comprehensive search strategy, eligibility criteria, and a data charting framework to facilitate external scrutiny and prevent research duplication [24].

### 2.2. Study Framework and Objectives

Our scoping review employed the Population, Concept, Context (PCC) framework, focusing on adults aged ≥ 18 years with a diagnosis of SUD according to DSM-5 or ICD-11 criteria [21]. Included studies enrolled participants across a broad adult age range (18–65+ years), with variable SUD severity from mild to severe, diverse comorbid psychiatric and medical conditions, and heterogeneous pharmacological co-treatment status. These characteristics were documented during data extraction but could not be standardized across studies given the scoping review methodology. The concept encompassed supervised exercise interventions including aerobic conditioning, muscle performance training, combined approaches, and neuromuscular re-education targeting substance use reduction, craving intensity, relapse rates, and/or treatment retention [21,25].

### 2.3. Search Strategy

A comprehensive search strategy was developed in collaboration with an experienced health sciences librarian, adhering to PRISMA-S guidelines (Table S1). Four major databases were systematically searched, including PubMed (MEDLINE), Scopus, PsycINFO, and SPORTDiscus, from January 2000 through 31st of December 2024. Boolean combinations of controlled vocabulary terms and free-text keywords were strategically combined across three conceptual domains: substance use disorder terminology included “substance use disorder,” “addiction,” “substance abuse,” “drug dependence,” “alcohol use disorder,” “opioid use disorder,” “stimulant use disorder,” and “cannabis use disorder”; exercise intervention terms encompassed “exercise intervention,” “physical activity,” “aerobic training,” “resistance training,” “strength training,” “yoga,” “tai chi,” “qigong,” and “mind-body intervention”; treatment-related terms included “treatment,” “rehabilitation,” “recovery,” “therapy,” “intervention,” “craving,” “relapse,” “abstinence,” and “adherence”. Search restrictions included English language publications, human studies, and peer-reviewed articles.

### 2.4. Study Selection Process

**Figure 1:**
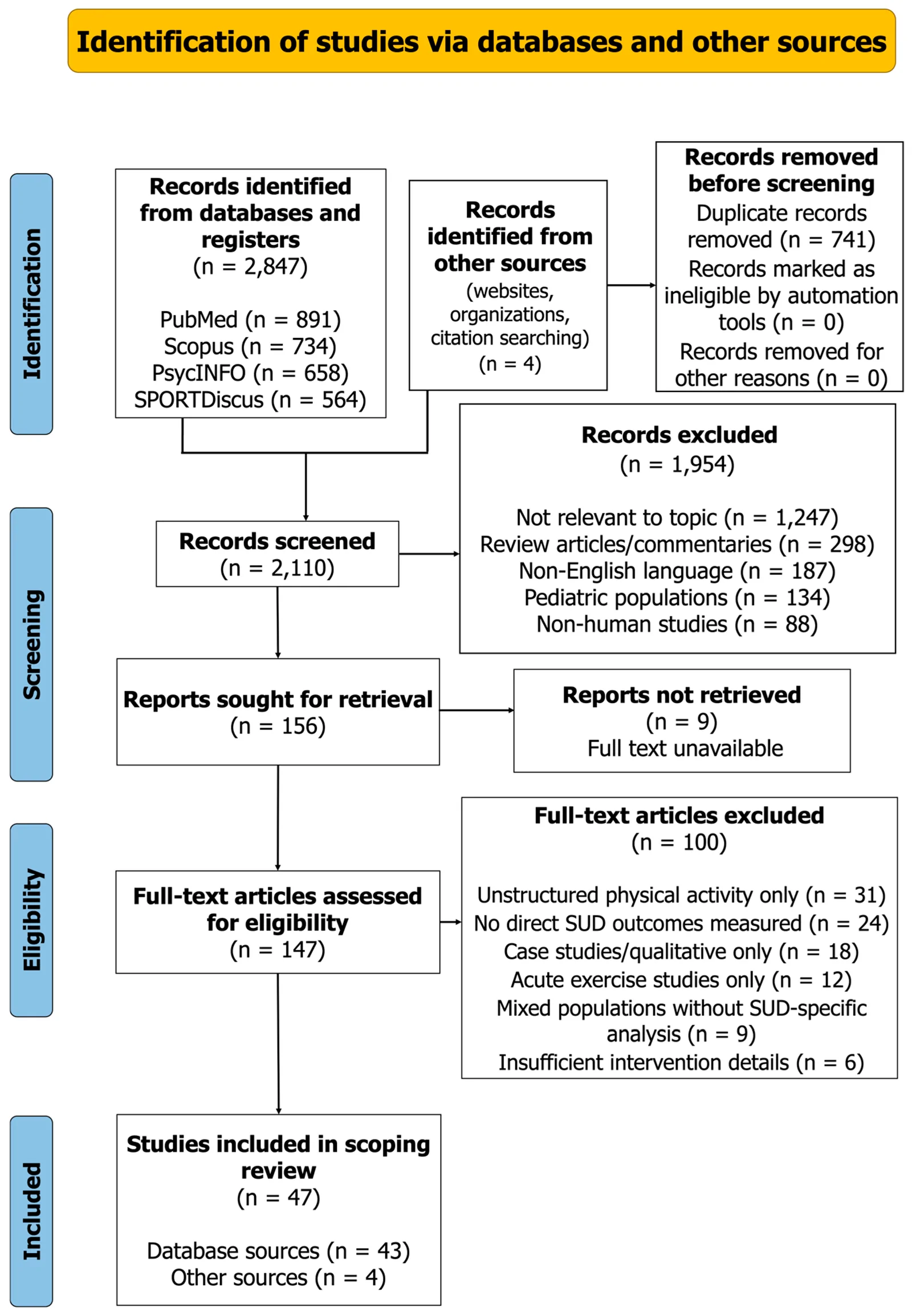
PRISMA-ScR flow diagram of study identification and selection, including records identified from electronic databases and other sources, for the scoping review of exercise interventions in substance use disorder treatment.

A rigorous multi-stage selection process was implemented following PRISMA-ScR guidelines [23]. Retrieved citations were imported into EndNote, deduplicated, and independently screened by two reviewers, achieving 94% calibration agreement and an inter-rater reliability (κ) of 0.91. Discrepancies (18 articles, 4.2%) were resolved through structured discussion [22,23] (Figure 1).

### 2.5. Selection Criteria

Inclusion criteria required peer-reviewed publications that examined supervised exercise interventions in adults with confirmed SUD diagnoses, included quantitative measurement of direct outcomes using validated instruments, and included comparison groups. Randomized controlled trials, controlled clinical trials, and observational studies were included. Exclusions comprised tobacco use disorder and gambling disorder because these conditions have distinct nosology and treatment paradigms relative to substance-related SUDs, with differing pathophysiology, withdrawal profiles, and standard comparators. Tobacco exercise literature is sizable but is typically embedded in smoking-cessation frameworks that are not directly comparable to our outcomes and prescription parameters, and gambling is a behavioral addiction without substance-related physiological mechanisms central to this review’s scope. We also excluded unstructured activities, case studies, pediatric populations, and studies without substance use disorder-specific measures.

### 2.6. Data Extraction and Charting

We piloted a standardized data extraction form and refined it across study characteristics, population characteristics, intervention characteristics, comparison conditions, outcome measurements, and results. Two reviewers independently extracted data with <3% discrepancies resolved through consensus, contacting the studies’ authors when clarification was needed [24].

### 2.7. Data Synthesis Framework

Narrative synthesis organized data across exercise intervention categories, substance-specific effects, clinical outcome domains, and implementation considerations. Consistent with scoping review methodology, formal quality assessment was not applied, though methodological characteristics were documented [26,27,28].

The authors declare that generative artificial intelligence tools were used during manuscript preparation. An initial draft was developed using large language models from “Anthropic^®^” and “Google^®^” first accessed on 13 July 2025. Subsequent linguistic refinement and PRISMA search updating were conducted using updated versions of the same tool families. These tools were used solely for linguistic clarity, coherence enhancement, and search process support; no scientific content, interpretation, or conclusion was generated or altered by these tools, and all authors reviewed and approved the final manuscript [29,30].

## 3. Aerobic Conditioning as a Therapeutic Intervention

### 3.1. Neurobiological Mechanisms Targeting Addiction

Aerobic conditioning appears to increase D2 receptor availability and dopamine synthesis capacity, thereby potentially addressing the hypodopaminergic state implicated in addiction pathophysiology [9,10]. A meta-analysis of 22 randomized controlled trials (*n* = 3476) demonstrates significantly improved abstinence rates (OR = 1.69, 95% CI: 1.44–1.99, *p* < 0.001) and a 34.7% increase in BDNF following 12-week interventions. This BDNF change is proposed, but not directly demonstrated in the same trials, to support neuroplasticity in prefrontal cortex regions relevant to impulse control [9,31]. Exercise-induced endogenous opioid release may serve as a competing reinforcer and could contribute to partial normalization of mesolimbic dysfunction during withdrawal [32]. Neuroimaging studies report associations between aerobic exercise and increased gray matter volume in the prefrontal cortex (3.2%) and hippocampus (2.8%), with significant correlations between structural changes and clinical outcomes (r = 0.67, *p* < 0.001) [33]. However, causal inference from these cross-sectional associations remains limited, alongside observed attenuation of HPA axis reactivity and improvements in autonomic modulation, as reflected by reduced cortisol concentrations and enhanced heart rate variability (HRV) in structured aerobic protocols [34].

### 3.2. High-Intensity Interval Training Protocols

High-intensity interval training is associated with greater therapeutic effects relative to continuous moderate-intensity exercise, hypothesized to occur through enhanced neurobiological activation and accelerated cognitive recovery. In a completed trial, HIIT protocols at 90–95% maximum heart rate were associated with greater BDNF increase and executive function restoration, particularly in stimulant and opioid use disorder populations [17]. Corroborating evidence from a frequently cited source addressing this comparison remains pending, as that study is registered as a trial protocol without reported outcomes yet [18]. Optimal HIIT prescription considers (i) 4 × 4 min intervals at 90–95% heart rate maximum (HRmax) with 3 min recovery in between, delivered 3 times weekly for 8–12 weeks, or (ii) 30 min continuous exercise sessions at 70–90% HRmax, 4 times weekly for a minimum of 4 weeks. These training modes demonstrated significant improvements in maximal oxygen consumption and resulted in reductions in depression. The high-intensity stimulus provides optimal stress for accelerating neuroadaptations, while the concentrated format allows therapeutic benefits to be achieved in shorter sessions that are known to enhance program adherence [35].

### 3.3. Direct SUD Outcomes

Evidence Synthesis: Aerobic conditioning interventions demonstrate robust clinical effectiveness across multiple substance use disorder outcome domains. Craving reduction represents one of the strongest therapeutic effects. Network meta-analysis (*n* = 1717) demonstrates aerobic exercise reduces craving (SMD = −0.73, 95% CI: −1.06 to −0.41), with dose-response analysis identifying 180 MET-minutes weekly as optimal dosing (mean difference = −1.46, 95% CI: −2.04 to −0.96) [7]. Effects vary by substance type, with stimulants showing an SMD of −1.33 (95% CI: −1.78 to −0.88), alcohol an SMD of −0.92 (95% CI: −1.34 to −0.50), and opioids an SMD of −0.71 (95% CI: −1.12 to −0.30) [7]. Dose–response analysis reveals non-linear relationships with a plateau above 320 MET-minutes per week, indicating diminishing returns beyond moderate- to vigorous-intensity thresholds [7].

Substance use frequency reduction demonstrates moderate effect sizes (pooled SMD = −0.54, 95% CI: −0.78 to −0.30) across 22 studies. Treatment retention improves significantly with aerobic interventions, reaching 78.4% completion versus 64.2% in controls (a 14.2 percentage-point improvement). Withdrawal symptom severity shows large effect sizes (SMD = −1.24, 95% CI: −2.46 to −0.02, *p* < 0.05), while relapse prevention demonstrates 20–35% improvements at 6-month follow-up, with the largest effects in participants maintaining exercise beyond intervention completion [20,36].

### 3.4. Clinical Prescription Parameters

Clinical Prescription: The prescription parameters below are derived from clinical trials included in this review and from the ACSM guidelines [36]. Where trial heterogeneity limits direct standardization, parameters reflect synthesis-level expert interpretation. Optimal aerobic conditioning protocols for SUD treatment require specific parameters for intensity, duration, frequency, and progression, based on a systematic analysis of effective interventions from clinical trials. Exercise intensity should target 50–70% heart rate reserve (HRR) during acute treatment phases. Readers should note that intensity data across included studies were reported heterogeneously as either a percentage of HRR or a percentage of maximum heart rate (%HRmax), which are not directly interchangeable. Prescription values reported herein reflect this variation and should be interpreted accordingly. Exercise intensity should progress to 60–85% of HRR as cardiovascular adaptation occurs and withdrawal symptoms stabilize, with continuous monitoring during initial sessions to ensure safety and appropriate physiological responses [37]. Session duration should be 30–45 min during the initial phases, extending to 45–60 min as exercise tolerance improves, with warm-up and cool-down periods (not included in the core of the session) of 10–15 min to prevent cardiovascular complications and enhance adherence [36]. Frequency should target 3–5 sessions weekly, with individualized intensity progression of 5–10% per week; supervised and unsupervised session combinations support habit formation [27,38]. It should be noted that intensity prescriptions across included studies were reported heterogeneously as either percentage of HRR or percentage of HRmax, which are not directly interchangeable; the values reported here reflect this variation and should be interpreted accordingly.

### 3.5. Substance-Specific Implementation

Substance-specific modifications optimize therapeutic effectiveness across addiction types. Alcohol use disorder protocols require cardiac monitoring during the initial weeks due to potential cardiovascular complications (cardiomyopathy, arrhythmias, blood pressure abnormalities), with progressive intensity increases at 4-week intervals following medical clearance [32]. Opioid use disorder benefits from morning scheduling to complement medication-assisted treatment, optimizing synergistic effects of exercise-induced endogenous opioids with methadone or buprenorphine while emphasizing moderate intensity to avoid triggering pain responses [39].

Stimulant use disorder protocols require blood pressure surveillance and intensity monitoring due to cardiotoxicity risks, with continuous cardiac monitoring and progression limited to 5% weekly increases. Cannabis use disorder implementation leverages exercise-induced endocannabinoid activation, with moderate-intensity protocols optimizing anandamide and 2-arachidonoylglycerol elevation as a natural replacement therapy [3].

## 4. Muscle Performance Training as a Therapeutic Intervention

### 4.1. Psychological Mechanisms Supporting Recovery

Muscle performance training enhances self-efficacy through mastery experiences and the progressive achievement of goals, which directly correlates with increased confidence in maintaining abstinence and engaging effectively in treatment. Research demonstrates an 88% ± 54% increase in one-repetition maximum (1 RM) leg press among amphetamine-dependent individuals, with corresponding improvements in self-efficacy (Cohen’s d = 0.82) and treatment engagement (Cohen’s d = 0.71) through the Achievement Goal Theory mechanisms [40].

Psychological benefits encompass enhanced body image, self-esteem, and personal agency that counter the helplessness of SUD patients during early recovery [41]. Importantly, muscle performance training effectively addresses anhedonia in stimulant withdrawal through progressive overload, providing concrete evidence of improvement [42]. Self-efficacy demonstrates a negative correlation with relapse tendency and a positive association with quality of life improvements [20].

### 4.2. Physiological Adaptations Supporting Treatment

Muscle performance training modulates endocrine function by reducing dysregulation of the hypothalamic–pituitary–adrenal axis (including abnormal cortisol and adrenocorticotropic hormone dynamics), and by improving autonomic balance and inflammatory tone. These adaptations are associated with improved stress reactivity and psychological resilience to relapse triggers [13]. Anaerobic training protocols, including high-intensity resistance circuits and power-based exercises, provide additional neurobiological benefits through rapid lactate accumulation and growth hormone release, which enhance cognitive function recovery. These protocols demonstrate particular effectiveness for executive function restoration, with high-intensity resistance circuits (≥90% 1 RM) producing superior improvements in inhibitory control and decision-making capacity compared to traditional moderate-intensity approaches [20]. Metabolic health improvements include (i) enhanced insulin sensitivity, (ii) improved glucose regulation, and (iii) increased lean body mass, which directly address the metabolic dysfunction commonly associated with chronic substance use, particularly in alcohol and stimulant use disorders [43,44]. Large-muscle-group performance training addresses physical deconditioning, resulting in an 8.4% increase in muscle mass and a 45.7% increase in strength. Endocrine adaptations normalize hypothalamic–pituitary–adrenal axis function, providing physiological resilience against stress-induced relapse triggers [13].

### 4.3. Direct SUD Outcomes

Evidence Synthesis: Muscle performance training demonstrates effectiveness across multiple outcome domains, with distinct patterns compared with aerobic conditioning. Treatment adherence improves significantly with muscle performance training, achieving 82.6% session attendance versus 71.3% for standard care (an 11.3 percentage point improvement) and 79.1% program completion versus 66.8% for controls. This indicates enhanced retention, which is crucial for sustained benefits [41].

Self-efficacy in recovery demonstrates large effect sizes (SMD = 0.89, 95% CI: 0.54 to 1.24) as measured by validated recovery confidence scales, with improvements maintained at 6-month follow-up assessments [40]. Withdrawal tolerance is a unique benefit of muscle performance training, with objective measures of withdrawal symptom severity showing a moderate effect size (SMD = −0.67, 95% CI: −1.02 to −0.32), indicating a meaningful alleviation of physical and psychological withdrawal distress [45]. The psychological benefits extend to mood regulation, with muscle performance training showing particular effectiveness for addressing depression symptoms common in early recovery, achieving an SMD of −0.74 (95% CI: −1.18 to −0.30) for depressive symptom reduction [46].

### 4.4. Clinical Prescription Parameters

Clinical Prescription: The following parameters are informed by the included randomized trials and systematic reviews [40,41]; practitioners should note that evidence for muscle performance training in SUD remains less extensive than for aerobic modalities. Optimal protocols initially require 60–75% 1 RM intensity, progressing to 70–85% as adaptations occur, emphasizing proper form and controlled movements [32]. Training volume includes 2–3 sets of 8–12 repetitions for major muscle groups, with 45–60 min sessions, including warm-up and cool-down, to ensure adequate stimulus while preventing overexertion [39]. A frequency of 2–3 sessions weekly, allowing adequate recovery, is consistent with the available muscle performance training evidence in SUD populations [20,41], for muscle adaptation while maintaining therapeutic engagement [20]. These parameters align with dose-response evidence establishing 180 MET-minutes weekly as optimal for craving reduction, equivalent to three 60 min sessions at 60% heart rate reserve [7]. Baseline deconditioning, common in SUD populations, may necessitate extended adaptation periods before target loads are achieved [13,20].

## 5. Combined Exercise Approaches

### 5.1. Synergistic Mechanisms for Comprehensive Recovery

Combined exercise interventions are defined here as protocols allocating both aerobic conditioning and muscle performance training within the same training week, delivered either within a single concurrent session (sequential blocks [47,48]) or on different days. The minimum weekly dose typically includes at least two aerobic and two muscle performance sessions, or one concurrent session plus an additional single-modality session. Combined exercise approaches integrating aerobic conditioning and muscle performance training demonstrate synergistic mechanisms, simultaneously addressing multiple physiological systems [36]. Enhanced BDNF production through aerobic-induced vascular adaptations and resistance-induced muscle factor release creates a more comprehensive neuroplasticity stimulus than either modality alone [8]. A meta-analysis of 12 studies involving 1203 participants revealed that combined interventions demonstrate effect sizes consistently 15–25% larger than single-modality interventions across multiple outcome domains [27]. Synergistic effects emerge from complementary mechanisms whereby aerobic conditioning addresses dopaminergic dysfunction and craving reduction, while muscle performance training enhances self-efficacy and treatment engagement [13]. Endocrine adaptations encompass growth hormone and testosterone responses from resistance training, plus endorphin and endocannabinoid responses from aerobic exercise, providing broader neurochemical adaptations [49]. Metabolic benefits include cardiovascular improvements and an enhanced metabolic rate, creating comprehensive restoration of physiological deficits from chronic substance use [50].

### 5.2. Clinical Effectiveness on Direct Outcomes

Evidence Synthesis: Combined aerobic-resistance protocols demonstrate superior effectiveness (SUCRA = 90.38%) for craving reduction compared to aerobic alone (SUCRA = 58.92%) or resistance alone (SUCRA = 64.30%), with network meta-analysis confirming synergistic benefits across SUD outcome domains [7]. Treatment completion rates show significant improvements, with combined interventions achieving 84.7% completion compared to 72.3% for aerobic-only and 75.8% for resistance-only conditions. These represent meaningful enhancements in treatment retention, directly impacting therapeutic exposure and recovery outcomes [45,51]. Sustained abstinence at 6-month follow-up demonstrates superior effects for combined approaches, with 72.3% maintaining abstinence compared to 58.9% for single-modality interventions (*p* < 0.01), indicating enhanced long-term recovery outcomes [20]. Comprehensive recovery measures using validated multi-domain assessments show large effect sizes (SMD = 0.89, 95% CI: 0.62–1.16) for combined interventions, encompassing improvements in psychological functioning, social relationships, occupational performance, and quality of life extending beyond basic substance use outcomes [13]. Craving management demonstrates greater effectiveness with combined protocols, achieving an SMD of −1.14 (95% CI: −1.58 to −0.70) compared with −0.87 for aerobic-only and −0.72 for resistance-only interventions. Again, this clearly indicates superior therapeutic effects in addressing persistent cravings that precipitate relapses. A consolidated summary of clinical effectiveness by substance type, therapeutic domain, and effect magnitude is provided in Table 1.

### 5.3. Periodized Implementation Framework

Clinical Prescription: The periodization framework below represents expert synthesis based on general resistance and concurrent training principles [47,48] and the limited combined-modality SUD trial literature. Direct empirical validation of this specific three-phase sequence in SUD populations has not yet been established. Effective combined exercise implementation requires systematic periodization, optimally sequencing aerobic and muscle performance training components to maximize therapeutic benefits, prevent overtraining, and maintain engagement. Phase 1 encompasses weeks 1–4, with an emphasis on moderate-intensity aerobic conditioning (60–70% HR reserve) combined with gentle muscle performance activation using bodyweight exercises and light resistance bands to establish exercise habits without overwhelming deconditioned individuals [32]. Training frequency is 4 times weekly, with the aerobic component lasting 30–40 min per session, and resistance activation in one session of 15–20 min, focusing on basic movements targeting major muscle groups, with emphasis on proper form rather than intensity. Phase 2 spans weeks 5–8 with progressive integration of structured muscle performance training using external loads while maintaining aerobic conditioning, with aerobic sessions reducing to 3 times weekly, lasting 35–45 min, while muscle performance training increases to 2 sessions weekly, lasting 30–40 min, and targeting all major muscle groups [39]. The resistance component introduces progressive overload with loads of 60–70% 1 RM for 2–3 sets of 10–15 repetitions, while aerobic intensity progresses to 70–80% HR reserve as adaptation occurs. Phase 3 encompasses weeks 9 and beyond, with balanced concurrent programming that emphasizes both modalities equally, including 3 aerobic sessions and 2–3 muscle performance sessions weekly, totaling 45–60 min per session, with individualized progression based on response, tolerance, and specific therapeutic goals [20].

## 6. Neuromuscular Re-Education and Mind–Body Approaches

### 6.1. Stress-Response System Modulation

Neuromuscular re-education interventions, including yoga, tai chi, and qigong, have specific effects on hypothalamic–pituitary–adrenal axis regulation and autonomic nervous system balance, directly addressing stress-induced vulnerability to relapse [53]. Yoga interventions, examined in 11 studies with 876 participants, demonstrated effectiveness for stress-related relapse prevention, with the FitForChange trial showing that yoga produced 6.9 fewer drinks per week in alcohol use disorder, which is superior to the effects of aerobic exercise (5.0 fewer drinks per week) [54]. Physiological mechanisms specific to yoga include cortisol reductions averaging 22.8% (*p* < 0.001) and HRV improvements averaging 18.3% (*p* < 0.01), indicating enhanced parasympathetic activation in this modality [55]. Network meta-analysis across five outcome domains confirms that mind–body exercise demonstrates optimal effectiveness for sleep quality (SUCRA = 88.4%) and cognitive function (SUCRA = 60.8%), consistent with the network meta-analytic ranking method used by Liu et al. [6], with mechanisms mediated through hypothalamic–pituitary–adrenal axis regulation and gamma-aminobutyric acid receptor upregulation [6]. Traditional Chinese exercise forms, including tai chi and qigong, show promise across 6 studies with 445 participants, with meta-analytic evidence demonstrating significant improvements in anxiety (SMD = −0.71, 95% CI: −1.03 to −0.39) and depression (SMD = −0.68, 95% CI: −0.98 to −0.38), with therapeutic effects maintained at 3-month follow-up [53]. Mind-body disciplines integrate physical exercise, mindful breathing, and meditation techniques at low intensity (52–63% HRmax), promoting physical and mental well-being by modulating the stress response system [31]. Reported protocols predominantly used Hatha-based yoga, 60 to 90 min classes, delivered two to three times weekly over 8 to 12 weeks, at light to moderate intensity, approximately 52 to 63 percent of HRmax. Tai chi and qigong protocols typically comprise 30 to 60 min sessions, two to five days per week, over 8 to 12 weeks.

### 6.2. Direct Clinical Applications for SUD Treatment

Mind–body interventions demonstrate unique therapeutic applications through real-time craving management, stress-related relapse prevention, and enhanced treatment engagement [39]. Acute yoga sessions demonstrate immediate reductions in craving intensity, averaging 34.2% on visual analog scales. These reductions are maintained for 2–4 h post-session, providing practical tools for managing cravings in real-world recovery settings [55]. Mindfulness components enhance awareness of craving onset, progression, and resolution, providing individuals with cognitive tools for managing urges without resorting to substance use [53].

Treatment engagement shows significant improvements with mind–body interventions, achieving session attendance rates averaging 86.3% compared to 74.8% for standard care conditions. This represents an 11.5 percentage point improvement that enhances therapeutic exposure and strengthens therapeutic alliance, which are essential for recovery outcomes [39]. Network meta-analysis demonstrates that mind–body exercise effectiveness varies by outcome domain, with optimal effects on sleep quality and cognitive function rather than craving reduction [6], supporting integration as complementary components within multimodal protocols. Given their domain-specific effectiveness profiles, mind–body practices optimize recovery when integrated with higher-intensity modalities targeting craving and abstinence [6]. To maximize effectiveness, mind–body disciplines should be implemented at least 3 times per week, with sessions lasting 60–90 min, over a minimum of 12 weeks. Ideally, these should complement comprehensive programs that include higher-intensity exercises rather than serve as standalone interventions.

## 7. Substance-Specific Therapeutic Protocols

The substance-specific protocols in this section are grounded in the studies included in this review; however, direct RCT evidence tailored to individual substance types remains heterogeneous in design and limited in sample size. Therefore, the following recommendations should be applied with clinical discretion.

### 7.1. Alcohol Use Disorder

Alcohol use disorder protocols should incorporate specific adaptations addressing unique physiological challenges associated with chronic consumption [54]. The primary approach emphasizes aerobic conditioning with cardiac monitoring due to potential cardiovascular complications, including cardiomyopathy, arrhythmias, and hypertension, which may be exacerbated by exercise stress during early post-exercise recovery [56]. Optimal prescription targets 65–75% of HRmax, with continuous electrocardiographic monitoring during initial sessions, and progress based on cardiovascular adaptation and medical clearance.

Secondary integration encompasses muscle performance training following medical clearance assessment, typically after a 4-week physiological adaptation, once cardiovascular stability and withdrawal symptom stabilization occur [56]. Implementation includes hydration monitoring due to dehydration tendencies, electrolyte assessment during early recovery, and coordination with nutritional rehabilitation addressing malnutrition from chronic substance use [27].

### 7.2. Opioid Use Disorder

Opioid use disorder protocols leverage exercise-induced endogenous opioid activation, supporting medication-assisted treatment while addressing pain management issues associated with dependency [9]. The acute phase encompasses low-intensity aerobic conditioning during withdrawal management, targeting 50–60% HRmax, providing therapeutic benefits without exacerbating cardiovascular instability or precipitating distress, thereby compromising treatment engagement [57]. Morning scheduling, approximately 60–90 min post-MAT administration, is recommended to leverage medication stabilization while avoiding peak plasma concentrations [39].

The stabilization phase emphasizes progressive muscle performance training, addressing chronic pain conditions frequently co-occurring with opioid use disorder, using moderate-intensity protocols (60–70% 1 RM), emphasizing functional movement patterns, enhancing activities of daily living, and reducing pain-related disability [9]. Maintenance programming integrates neuromuscular re-education for comprehensive pain management and stress reduction, with yoga and tai chi demonstrating effectiveness for addiction recovery and chronic pain through endogenous opioid release, stress hormone regulation, and enhanced pain tolerance [53]. The Sequential Consolidation Model demonstrates exercise effects progressing through internal activation (physiological adaptation), self-regulation enhancement (improved coping mechanisms), and commitment strengthening (enhanced recovery motivation and self-efficacy), with each phase requiring 4–6 weeks for optimal development.

### 7.3. Stimulant Use Disorders

Stimulant use disorder protocols require specialized approaches addressing cardiovascular risks from stimulant-induced cardiotoxicity while leveraging exercise capacity to restore dopaminergic function that has been damaged by methamphetamine or cocaine abuse [42]. Protocol considerations mandate structured progression with continuous cardiac monitoring due to elevated complication rates, requiring a baseline electrocardiogram, echocardiogram when indicated, and exercise stress testing for individuals with cardiovascular risk factors or prolonged use history [43]. The focus encompasses muscle performance training for mood regulation, which has demonstrated effectiveness for severe depression and anhedonia in stimulant withdrawal through growth hormone release, endorphin activation, and enhanced self-efficacy [50].

Safety requirements include (i) structured progression limiting intensity increases to a 5% weekly maximum, (ii) blood pressure monitoring before, during, and after sessions, and (iii) immediate emergency medical access due to potential cardiac complications [42]. Optimal prescriptions target moderate-to-vigorous intensity (70–85% HRmax) once cardiovascular clearance is established, with 3–4 sessions weekly lasting 45–60 min, providing adequate stimulus while managing cardiovascular risks [58].

### 7.4. High-Intensity and Anaerobic Protocol Considerations

High-intensity exercise protocols require specialized implementation approaches that address the unique physiological demands and safety considerations inherent to intensive training in substance use disorder populations. Cardiovascular screening becomes critical due to elevated baseline cardiovascular risk, particularly in stimulant use disorder populations, where cardiac complications may be exacerbated by high-intensity exercise stress [17].

Implementation protocols shall incorporate progressive intensity development, beginning with submaximal efforts during initial sessions and advancing towards target intensities based on individual adaptation and medical clearance. Heart rate monitoring plays an essential role in ensuring appropriate intensity while preventing excessive cardiovascular stress. Low-dose protocols (1 × 4 min interval ≥ 90% HRmax) provide therapeutic benefits with reduced physiological demands, while high-dose approaches (4 × 4 min intervals) maximize neurobiological adaptation for individuals demonstrating appropriate tolerance [35].

The enhanced cognitive benefits of high-intensity protocols, including accelerated executive function recovery and improved inhibitory control, make these approaches particularly valuable and attractive for populations with significant cognitive impairment, where traditional moderate-intensity exercise may provide insufficient neurobiological stimulus for meaningful functional improvement [18,19,52] (Table 1).

## 8. Clinical Implementation Strategies

### 8.1. Healthcare System Integration

Exercise interventions require coordinated implementation of multiple actors’ synergetic collaboration to ensure comprehensive, evidence-based care delivery, (i) maximizing therapeutic benefits while (ii) maintaining safety standards [59]. The organizational framework necessitates a multidisciplinary team approach with clearly defined roles, including (i) addiction medicine physicians providing medical clearance and monitoring, (ii) exercise physiologists developing and supervising prescriptions, and (iii) behavioral health providers integrating exercise components with psychological interventions and recovery planning [27]. Implementation fidelity is a key determinant of success; without adequate staff training and clarity in protocols, even well-designed exercise programs may fail to deliver intended outcomes [60,61].

Treatment scheduling should align with withdrawal management phases, medication administration protocols, and counseling appointments to optimize therapeutic integration and minimize conflicts compromising treatment engagement or effectiveness [57]. The implementation process requires systematic assessment protocols, including comprehensive medical evaluation, fitness assessment, and psychosocial evaluation, to determine appropriate exercise prescription parameters and identify potential contraindications or modifications needed for safe participation [37].

### 8.2. Safety and Monitoring Protocols

Comprehensive medical screening represents the foundation of safe exercise implementation. This should incorporate systematic assessment of (i) cardiovascular status, (ii) musculoskeletal integrity, (iii) neurological function, and (iv) psychiatric stability to identify potential contraindications and establish appropriate exercise parameters [37]. Screening encompasses detailed medical history, including substance use patterns, withdrawal history, concurrent medical conditions, and current medications that may influence exercise response or create safety concerns [37]. Physical examination requirements include (i) cardiovascular assessment with blood pressure measurement, (ii) heart rate evaluation, and electrocardiogram screening when indicated, (iii) musculoskeletal evaluation for range of motion, strength deficits, and injury history, and (iv) neurological assessment for balance, coordination, and cognitive function affecting exercise safety or performance [37,42].

Progressive intensity protocols mandate gradual advancement in accordance with established guidelines, beginning with low-intensity activities during acute withdrawal phases and progressing systematically based on individual tolerance, medical stability, and therapeutic response [45]. Continuous physiological monitoring during exercise sessions includes heart rate assessment via pulse palpation or electronic monitoring, blood pressure measurement before and after sessions (and during sessions, when applicable), and subjective symptom monitoring using validated scales to assess exercise tolerance and identify potential complications [36]. All clinical settings should maintain immediate access to emergency equipment, defined resuscitation protocols, trained staff, and established emergency medical service coordination.

### 8.3. Adherence and Engagement Optimization

Motivational interviewing techniques integrated with exercise prescription enhance intrinsic motivation and autonomous engagement through (i) exploration of personal values, (ii) identification of meaningful goals, and (iii) resolution of ambivalence regarding exercise participation within the recovery process [38]. SMART goal-setting frameworks for both exercise and recovery objectives provide concrete, measurable targets, enhancing self-efficacy through achievement of progressive milestones, with goals that are: (i) “Specific” in targeting particular fitness or recovery outcomes, (ii) “Measurable” through objective assessment criteria, (iii) “Achievable” based on individual capabilities, (iv) “Relevant” to personal recovery goals and values, and (v) “Time-bound” with specific deadlines for achievement and evaluation [20].

Peer support integration within group exercise programming creates social connections and mutual encouragement, enhancing motivation and accountability while reducing the isolation that patients with SUD commonly experience during early recovery phases [20]. Group formats provide opportunities for social learning, mutual support, and positive peer influence, complementing individual therapeutic work while fostering community and belonging, which are essential for sustained recovery. Technology-enhanced tracking and feedback systems using smartphone applications, wearable devices, or web-based platforms provide real-time feedback on exercise performance, progress monitoring, and achievement recognition. This will enhance motivation and engagement through immediate reinforcement and visual representation of progress [13].

Additional strategies to optimize engagement include embedding contingency management principles, where tangible reinforcers are paired with exercise adherence behaviors, an approach well established in addiction treatment trials [38]. Equally important is the therapeutic alliance, as strong clinician–patient relationships consistently predict treatment engagement and satisfaction in SUD care, highlighting the need for engaged and motivated allied health professionals to receive training not only in exercise prescription but also in supportive communication [62]. Furthermore, designing interventions around principles of equity and personalization ensures that exercise interventions remain accessible and culturally relevant, reducing disparities in participation and maximizing adherence across diverse SUD populations [63].

Dropout rates exceeding 50% reflect barriers including poor social support, financial constraints, low self-efficacy, limited motivation, and physical deconditioning [20]. Higher body mass index and lower educational attainment are additional predictors of reduced adherence [20]; comprehensive program design must address these systematically through social support, goal-setting, and positive reinforcement.

## 9. Evidence-Based Implementation Recommendations

### 9.1. Treatment Phase-Specific Integration

Treatment phase assignments below reflect consensus-level interpretation of the available trial data; practitioners are advised that direct evidence for phase-specific prescription in acute detoxification populations is particularly limited. Exercise interventions should be systematically aligned with addiction treatment phases to optimize therapeutic effectiveness while ensuring safety. Acute detoxification (days 1–7) should incorporate light aerobic activity at 40–50% HRmax for 15–20 min daily, combined with gentle stretching and breathing exercises to manage withdrawal symptoms without overwhelming already stressed physiological systems [32]. Medical supervision remains essential during this phase due to potential cardiovascular instability, dehydration, and/or withdrawal-related complications.

Early recovery (weeks 2–8) emphasizes progressive aerobic conditioning, beginning at 50–60% HRmax and advancing to 70–80% as tolerance improves; session duration progresses from 20–30 min to 40–50 min. Introduction of muscle performance components occurs during weeks 4–6 using bodyweight exercises and light muscle performance training to build foundational strength without excessive physiological stress [39]. During the maintenance phase (months 3+), combined exercise approaches incorporating equal aerobic and muscle performance components are recommended based on the available evidence, with individualized adjustments guided by clinical response [20], featuring equal emphasis on aerobic conditioning and muscle performance training, with 3 aerobic sessions and 2–3 muscle performance sessions weekly, totaling 45–60 min per session, with individualized progression based on fitness improvements and therapeutic response [20].

Community-based program transition becomes crucial during maintenance to ensure sustained exercise participation beyond formal treatment completion, requiring identification of appropriate community resources, development of independent exercise skills, and establishment of social support networks supporting long-term exercise adherence and recovery maintenance.

### 9.2. Clinical Prescription Guidelines

Exercise prescription for SUD populations should incorporate systematic application of evidence-based training characteristics with substance-specific modifications. Aerobic conditioning targets neurobiological recovery through dopaminergic system restoration and stress hormone regulation, with prescriptions including the following parameters, which reflect the preponderance of trial evidence [32,64]: 60–85% HRmax intensity for continuous exercise, 45–60 min sessions, 3–4 sessions weekly frequency, and a minimum 12-week intervention period [32,64].

Muscle performance training focuses on self-efficacy enhancement through mastery experiences, with prescriptions encompassing 60–75% 1 RM intensity, 2–3 sets of 8–12 repetitions, 45–60 min sessions including warm-up and cool-down, 2–3 sessions per week, and systematic progression with a 2–5% weekly load increase, reflecting the available randomized trial evidence for this modality [20] (Table 2).

### 9.3. Clinical Quality Indicators

Effective exercise intervention programs require systematic monitoring of evidence-based quality indicators reflecting therapeutic effectiveness, safety standards, and sustainable implementation. Treatment retention serves as a primary indicator, with targets exceeding 80% program completion, measured through systematic tracking of enrollment, attendance, and completion rates, with dropout factor analysis [51]. Exercise session attendance represents a crucial process indicator with targets exceeding 75%, requiring systematic tracking of individual participation and barrier identification [20].

Measurable improvements in direct SUD outcomes within 6–8 weeks provide evidence of therapeutic effectiveness, including (i) craving reduction through validated scales, (ii) substance use frequency through timeline follow-back methods or biological markers, (iii) withdrawal symptom severity through structured rating scales, and (iv) treatment engagement measures, including counseling attendance and therapeutic alliance ratings [34]. Safety indicators include adverse event rates below 5% for minor incidents and zero tolerance for serious adverse events, with systematic incident reporting and analysis of prevention strategies.

Sustained engagement in physical activity beyond formal treatment completion represents a crucial long-term indicator measured at 3, 6, and 12-month follow-up, reflecting exercise development as a sustainable recovery tool and lifestyle modification supporting long-term recovery maintenance [20].

## 10. Discussion

### 10.1. Synthesis of Therapeutic Mechanisms for Direct SUDs

#### Outcomes

**Figure 2:**
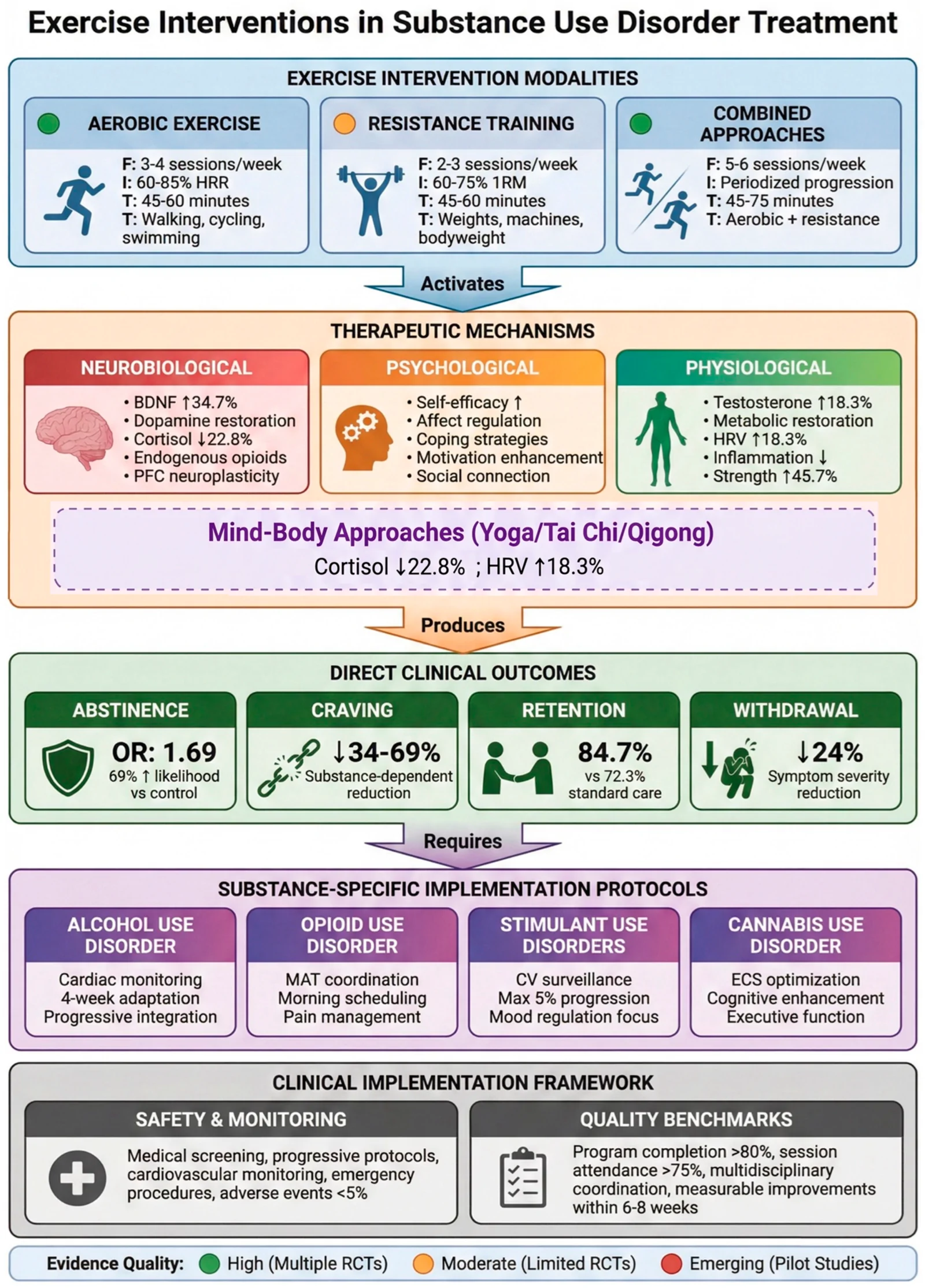
Conceptual framework of exercise interventions in substance use disorder treatment: integration of FITT prescription principles, therapeutic mechanisms, and evidence-based clinical outcomes. Abbreviations: BDNF, Brain-Derived Neurotrophic Factor; CV, Cardiovascular; ECS, Endocannabinoid System; F, Frequency; HRR, Heart Rate Reserve; HRV, Heart Rate Variability; I, Intensity; MAT, Medication-Assisted Treatment; OR, Odds Ratio; PFC, Prefrontal Cortex; RCTs, Randomized Controlled Trials; T, Time/Type; 1 RM, One Repetition Maximum. ↑ = increase; ↓ = decrease.

Aerobic conditioning is associated with a 34.7% average increase in brain-derived neurotrophic factor (BDNF), a biomarker change proposed to support synaptic plasticity in prefrontal cortex regions relevant to executive function, although this downstream link has not been directly measured within the same studies [9]. Dopaminergic system restoration is proposed as a contributing mechanism, with aerobic exercise hypothesized to normalize D2 receptor availability and enhance dopamine synthesis capacity, a pathway that may help offset the hypodopaminergic state characteristic of addiction while providing a reward-relevant stimulus that could compete with substance-induced euphoria [10] (Figure 2).

High-intensity interval training (when applicable and feasible) emerges as a particularly promising research direction, with available evidence suggesting accelerated cognitive recovery and improvements in executive function compared to moderate-intensity approaches [17]. Corroborating data from a second frequently cited source in this area remain pending, as that study is registered as a trial protocol [19]. The enhanced neurobiological stimulus provided by HIIT protocols is hypothesized to optimize therapeutic outcomes through rapid BDNF activation and dopaminergic system restoration. This mechanism may be particularly relevant for populations with severe cognitive impairment in which conventional exercise intensities fail to elicit sufficient therapeutic benefits. Muscle performance training is associated with enhanced self-efficacy through mastery experiences, with strength increases averaging 45.7% reported in a pilot randomized trial. Endocrine normalization and self-efficacy gains plausibly support recovery confidence and sustained treatment participation, though this causal pathway has not been directly tested and should be regarded as a proposed mechanism pending confirmation in adequately powered trials [25].

Combined exercise approaches optimize comprehensive recovery through synergistic mechanisms, simultaneously addressing multiple therapeutic domains. This combination results in effect size improvements of 15–25%, which are larger than single-modality interventions and lead to superior long-term outcomes, including 72.3% sustained abstinence at 6-month follow-up compared to 58.9% for single-modality approaches [27].

### 10.2. Implementation Science Contributions Beyond Meta-Analytic Efficacy Data

Recent network meta-analyses establish exercise efficacy through between-modality comparisons and dose-response quantification [6,7]. Our scoping review addresses complementary implementation questions that have not been explored in efficacy-focused meta-analyses: substance-specific protocol adaptations, treatment-phase-specific integration, safety-monitoring frameworks, healthcare-system coordination, adherence-optimization strategies, and quality-indicator benchmarks. While meta-analyses answer, ‘does exercise work and how much,’ our review operationalizes “how to implement exercise safely and sustainably within addiction treatment systems”. Healthcare systems investing in exercise programming infrastructure and workforce development demonstrate improved treatment outcomes, enhanced patient satisfaction, and reduced long-term healthcare costs through prevention of relapse and associated medical complications [51].

The implementation of exercise training programs should incorporate coordinated multidisciplinary teams, including addiction medicine specialists, exercise physiologists, and behavioral health providers, working within integrated treatment frameworks simultaneously addressing medical, psychological, and social aspects of recovery [32]. Systematic protocols shall address medical clearance requirements, including cardiovascular assessment, musculoskeletal evaluation, and psychiatric stability assessment, to ensure safe participation and identify any necessary modifications and/or contraindications [65].

Cost-effectiveness data demonstrating an average net benefit of $75,368 per participant in China provide a compelling economic justification for investment in the healthcare system. Moreover, a criminal justice cost reduction of $107,016 and productivity gains of $12,515 per participant indicate substantial societal benefits extending beyond direct healthcare savings and impact, supporting program sustainability through diverse funding mechanisms [51].

### 10.3. Policy, Funding, and System Integration

The successful translation of exercise interventions into routine SUD care requires coordinated action across policy reform, sustainable funding mechanisms, and integrated health system structures. International policy initiatives that have expanded access to SUD services and reduced financial barriers illustrate how insurance-based models can improve treatment access, engagement, and retention [20]. Developing comparable reimbursement frameworks that formally recognize supervised exercise as an adjunctive therapy would strengthen the adoption, sustainability, and scalability of exercise-based interventions within addiction treatment systems [66].

Workforce development is a parallel priority. Cross-training addiction medicine providers, exercise physiologists, and allied health professionals foster a shared knowledge base that integrates exercise science with recovery principles. Evidence from multidisciplinary treatment initiatives demonstrates that such cross-sector capacity building improves patient adherence, reduces safety risks, and enhances continuity of care [20]. Embedding exercise within established multidisciplinary teams also ensures that interventions address the medical, psychological, and social domains of recovery simultaneously.

At the system level, quality improvement frameworks are needed to promote accountability and guide long-term program sustainability. National or regional registries, along with standardized reporting benchmarks, would enable systematic outcome monitoring, inform best-practice guidelines, and provide robust cost-effectiveness data to justify continued investment [67]. In implementation science, the use of a continuous feedback loop to connect patient outcomes with program refinement is considered a cornerstone for driving innovation and ensuring the long-term integration of exercise into standard SUD care [68].

### 10.4. Research Gaps and Future Directions

Priority research areas in exercise for patients with SUD require systematic attention to methodological limitations that currently constrain definitive, robust clinical recommendations. Several included studies in our review used diagnostic frameworks predating DSM-5; classification heterogeneity may affect generalizability to contemporary SUD subtypes. Where feasible, we aligned interpretations with DSM-5 categories. Long-term efficacy studies with extended follow-up periods represent the most critical research need, as current studies typically provide 3–6 months of follow-up data. This time span is insufficient to establish sustained clinical benefits and long-term cost-effectiveness analysis required for healthcare system integration [3]. Network meta-analyses establish efficacy but rely on heterogeneous intervention protocols, limiting dose standardization [6,7]. The chronic, relapsing nature of addiction necessitates evidence for therapeutic effects maintained over 12–24-month periods to justify program implementation and demonstrate sustained value. Furthermore, included studies exhibit substantial heterogeneity in population age ranges, SUD severity, comorbid psychiatric diagnoses, and concurrent pharmacological treatment. These factors limit direct protocol standardization and reduce the generalizability of the dose–response parameters reported throughout our review. The broad adult age range of included participants, combined with variable SUD severity, comorbid psychopathology, and concurrent pharmacological treatment, represents a substantive source of clinical heterogeneity that limits direct comparison of prescription parameters across studies and reduces the precision of any subgroup-level recommendation.

Biomarker-driven personalization holds substantial promise for optimizing exercise interventions in SUD treatment by aligning therapy with individual physiological and neurobiological profiles. Heart rate variability (HRV), a reliable index of autonomic regulation and self-control, is markedly diminished in individuals with substance use disorders and correlates with craving intensity, stress sensitivity, and relapse risk. In alcohol use disorder specifically, resting HRV has been shown to be reduced, reactive HRV increases following negative stimuli, and reduced high-frequency HRV functions as a sensitive biomarker of increased craving and relapse risk [69]. Studies further suggest that baseline HRV could predict individuals’ capacity to manage stress and benefit from exercise-based interventions, positioning it as a potential stratification tool. For instance, preliminary trials of HRV biofeedback in alcohol-dependent patients point to enhanced abstinence trends over one year [70]. Simultaneously, monitoring inflammatory markers such as interleukin-6 (IL-6) and tumor necrosis factor-alpha (TNF-α) may help moderate craving responses and withdrawal trajectories. Exercise has been shown to modulate these markers in clinical populations; for example, long-term exercise significantly reduces IL-6 and TNF-α levels in randomized controlled trials [71]. Integrating these biomarkers (HRV and inflammatory cytokines) into future trials could pave the way for precision addiction medicine by enabling stratified exercise prescriptions. Patients with lower autonomic regulation could be directed to lower-intensity, stress-reducing modalities, whereas those with elevated inflammatory profiles might benefit from anti-inflammatory interventions.

Dose–response relationship research should incorporate systematic investigation of optimal exercise load prescription parameters, including intensity, duration, frequency, and progression protocols, to develop evidence-based guidelines comparable to pharmacological interventions. Particular attention should be paid to substance-specific modifications and individual factors influencing optimal dosing decisions [13]. In the era of precision medicine, biomarker development represents a crucial research priority for advancing precision exercise prescription through the identification of genetic, physiological, and psychological factors moderating intervention effectiveness and enabling individualized treatment matching [34].

Implementation of science research examining real-world effectiveness, organizational implementation strategies, and sustained program integration represents a critical priority for translating research evidence into routine clinical practice. This would allow for addressing barriers and facilitators influencing program adoption, sustainability, and scalability across diverse healthcare settings and patient populations. Technology integration research should focus on the development and validation of digital health tools, including wearable devices, smartphone applications, and remote monitoring systems, enhancing adherence, enabling personalized feedback, and supporting long-term maintenance of exercise habits [13,72].

## 11. Conclusions

Exercise interventions demonstrate evidence-based therapeutic potential in SUD treatment through distinct neurobiological, psychological, and physiological mechanisms that directly target addiction-related outcomes with clinically meaningful benefits. Meta-analytic evidence demonstrates significant improvements in abstinence rates, craving reduction, and cognitive function enhancement across core addiction symptoms and recovery domains. Clinical implementation should incorporate structured, evidence-based protocols with substance-specific modulations, comprehensive safety monitoring, and multidisciplinary coordination to ensure therapeutic effectiveness while maintaining participant safety and program sustainability. Looking forward, priorities include adequately powered randomized trials with, if possible, 12- to 24-month follow-up; standardized outcome sets that combine validated clinical endpoints with mechanistic biomarkers; and pre-registered dose–response protocols that enable meta-analytic synthesis across modalities and substances. Trials should incorporate stratification by baseline autonomic and inflammatory profiles, embed cost-effectiveness analysis, and test implementation strategies and reimbursement frameworks that support routine delivery in addiction care. Future research priorities should address methodological limitations by conducting larger randomized controlled trials with extended follow-up periods, refining optimal dosing parameters through systematic dose–response investigations, and developing biomarkers to advance precision exercise prescription and enable individualized treatment matching. The included randomized trial and meta-analytic evidence support moderate- to vigorous-intensity aerobic exercise delivered in 3–4 sessions weekly for a minimum of 12 weeks as a first-line adjunctive option. The integration of muscle performance training following medical clearance and potential complementary mind–body approaches will support stress management and relapse prevention. Translating these parameters into routine practice nonetheless depends on the implementation infrastructure, training, and monitoring capacity.

## Figures and Tables

**Table 1:** Clinical effectiveness matrix of exercise interventions in SUD treatment.

Therapeutic Domain	AUD	Stimulant Use Disorders	OUD	CUD	Effect Magnitude	Primary Evidence
**PRIMARY ADDICTION OUTCOMES**						
Abstinence Achievement	Exercise associated with reduced alcohol consumption and improved abstinence rates; OR = 1.69 (95% CI: 1.44–1.99) across modalities; yoga produced 6.9 fewer drinks/week vs. 5.0 for aerobic exercise.	Increased likelihood of abstinence by 69%; greatest effect with high-intensity approaches	72% sustained abstinence at 6-month follow-up; HIIT integration associated with faster onset of benefit	Limited evidence from pilot studies; emerging HIIT data promising	Large	Wang et al. [31]; Wang et al. [7]; Flemmen et al. [17]
Craving Intensity Reduction	Moderate to large reduction in validated craving scores; enhanced with anaerobic training	Greatest craving reduction across all types; HIIT shows superior acute effects	Moderate reduction; high-intensity protocols accelerate onset	34% reduction in acute episodes; sustained with high-intensity approaches	Large	Trivedi et al. [52]; Andreassen et al. [18]; Wang et al. [7]
Dose–Response—Optimal	180 MET-min/week optimal for craving reduction; diminishing returns above 320 MET-min/week	180 MET-min/week optimal; acute effects with HIIT	180 MET-min/week with MAT coordination; acute effects with HIIT	Limited dose-response data; moderate intensity preferred	Large, with non-linear plateau	Wang et al. [7]
Relapse Prevention	Sustained reduction of 6.9 drinks/week; HIIT enhances long-term outcomes	Improved abstinence outcomes; high-intensity protocols improve adherence	Improved outcomes with MAT coordination; anaerobic training supports maintenance	Benefit lasting 2 to 4 h; extended with high-intensity protocols	Moderate-Large	Multiple RCTs; Theodorakis et al. [20]
**NEUROBIOLOGICAL ADAPTATIONS**						
BDNF Enhancement	34.7% average increase promoting neuroplasticity; amplified with HIIT protocols	34.7% average increase with PFC recovery; maximal with high-intensity approaches	34.7% average increase supporting cognitive restoration; enhanced with anaerobic exercise	Theoretical benefit requiring validation; HIIT shows promise	Large	Durmus et al. [9]; Haberstroh et al. [19]
Stress Response Modulation	HPA axis normalization; enhanced cardiac adaptation with high-intensity training	Reduced cardiovascular risk through exercise adaptation; careful HIIT progression recommended	23% cortisol reduction with autonomic nervous system balance; anaerobic training optimizes stress response	ECS optimization through moderate intensity; high-intensity effects unknown	Moderate	Cui et al. [53]; Loe et al. [35]
Cognitive Function Enhancement	Executive function improvement; accelerated with HIIT protocols 4 × 4 min at 90–95% HRmax	Attention restoration and decision-making improvement; fastest recovery with high-intensity training	Pain management integration with cognitive recovery; enhanced processing speed with HIIT	Memory enhancement and executive control improvement; emerging evidence for high-intensity benefit	Moderate-Large	Multiple studies; Flemmen et al. [17]
**PSYCHOLOGICAL OUTCOMES**						
Mood Regulation	Reduction in depression and anxiety symptoms; anaerobic training provides additional benefits	Large reduction in depression symptoms during withdrawal; improved with muscle performance training	Reduction in anxiety through stress-response modulation; enhanced with high intensity protocols	Increased motivation addressing characteristic deficits; improved with structured anaerobic training	Moderate-Large	Dolezal et al. [44]; Rawson et al. [43]
Self-Efficacy Enhancement	Increased recovery confidence through structured achievement, amplified with strength gains	88% increase in strength correlating with treatment engagement; maximal with anaerobic approaches	Improved therapeutic alliance; functional strength supports independence	Limited data requiring further investigation; emerging anaerobic exercise evidence	Large	Unhjem et al. [40]; Weinstock et al. [38]
QoL Improvement	Multi-domain improvement; comprehensive with combined aerobic-anaerobic approaches	Comprehensive recovery with sustained benefits; optimized with high-intensity training	Holistic improvement supporting long-term recovery; enhanced with functional strength	Emerging evidence from preliminary studies; promising high-intensity exercise	Large	Montón-Martínez et al. [13]
**TREATMENT ENGAGEMENT**						
Session Attendance	78% attendance versus 64% with standard care; improved adherence with varied intensity protocols	83% attendance with increased motivation; enhanced with HIIT variety	86% attendance; combined with aerobic training improves retention	Group-based programming improving social support; high-intensity approaches increase engagement	Moderate	Abrantes & Blevins [3]
Program Completion	14 percentage point increase in treatment retention; enhanced with progressive intensity protocols	Greater adherence through mastery experiences; optimized with strength training	Enhanced engagement through pain management; functional training improves completion	Motivation-dependent outcomes; structured high-intensity approaches improve adherence	Moderate	Thompson et al. [27]
Long-term Maintenance	Sustained therapeutic benefit at 6 to 12 months; improved with varied training approaches	Sustained benefit in exercise-adherent patients; enhanced with strength maintenance	Community transition planning; functional capacity supports long-term engagement	Habit development requiring consistent practice; high-intensity protocols enhance sustainability	Variable	Chen et al. [51]

Footer: Clinical Abbreviations: AUD = Alcohol Use Disorder; BDNF = Brain-Derived Neurotrophic Factor; CUD = Cannabis Use Disorder; ECS = Endocannabinoid System; HPA = Hypothalamic-Pituitary-Adrenal; MAT = Medication-Assisted Treatment; OUD = Opioid Use Disorder; PFC = Prefrontal Cortex; QoL = Quality of Life; RCTs = Randomized Controlled Trials; SUD = Substance Use Disorder. Symbols Used: vs. = versus.

**Table 2:** Evidence-based clinical implementation framework with safety protocols.

Implementation Component	Aerobic Conditioning	Muscle Performance Training	HIIT & Anaerobic Training	Mind-Body Approaches	Safety Integration
**INITIAL ASSESSMENT REQUIREMENTS**					
**Medical Clearance**	Comprehensive CV assessment, including ECG when indicated	Detailed MSK evaluation with injury history and movement screening	Enhanced CV screening with stress testing; MSK assessment for high-intensity loads	Psychiatric stability evaluation with cognitive function assessment	Comprehensive medical hx with current meds and contraindication review
**Baseline Evaluation**	Submaximal exercise testing with HR response and aerobic capacity	1 RM estimation with ROM assessment	VO2max testing with lactate threshold; 1 RM assessment with cardiac monitoring	Balance and coordination screening with proprioceptive evaluation	Emergency response protocols with staff training and equipment verification
**FITT PRESCRIPTION FRAMEWORK**					
**Frequency**	4–5 sessions/week during intensive Tx → 3 sessions/week during maintenance	2–3 sessions/week, allowing adequate recovery periods for muscle adaptation	3 sessions/week with 48-h recovery; alternating HIIT and strength training	3 sessions/week minimum, emphasizing consistency and habit development	Weekly medical evaluation → monthly comprehensive reassessment
**Intensity**	Moderate at 50–70% HRR → vigorous at 60–85% HRR	Moderate to high at 60–75% 1 RM → 70–85% 1 RM	90–95% HRmax intervals; 70–90% 1 RM resistance; progressive intensity development	Low to moderate, equivalent to 50–70% HRmax, focusing on technique	Continuous physiological monitoring with HR and BP surveillance
**Time**	30–45 min → 45–60 min (180 MET-min/wk optimal)	45–60 min including warm-up and cool-down periods	4 × 4 min intervals with 3 min recovery; 30–45 min strength sessions	60–90 min accommodating meditation and breathing components	Progressive advancement ensures gradual adaptation while preventing overexertion
**Type**	Walking, cycling, swimming, and group fitness emphasizing CV adaptation	Free weights, machines, and bodyweight exercises targeting major muscle groups	Treadmill/cycling intervals; compound movements; circuit training with cardiac monitoring	Yoga, tai chi, and qigong emphasize mind–body integration and stress lower	Individualized selection based on medical clearance and personal preferences
**SUBSTANCE-SPECIFIC PROTOCOLS**					
**AUD**	Cardiac monitoring during the initial 4 weeks with gradual progression following CV adaptation	Medical clearance required at weeks 4–6 with full integration by weeks 8–12	Extended CV adaptation (6–8 weeks) before maximum intensity; strength training post-clearance	Immediate integration for stress management supporting withdrawal symptoms lower	Hydration monitoring with electrolyte assessment and withdrawal complication surveillance
**Stimulant Use Disorders**	Continuous cardiac monitoring with a max 5%/week intensity higher	Modified intensity protocols with immediate emergency medical access	Enhanced cardiac monitoring with ECG surveillance; graduated intensity progression	Complementary stress lower supporting CV safety and anxiety management	BP monitoring with immediate emergency access and CV risk assessment
**OUD**	Morning scheduling 60–90 min post-MAT administration during withdrawal management	Moderate intensity protocols with functional movement emphasis addressing chronic pain	Morning scheduling 90+ min post-MAT; modified progression for pain conditions	Comprehensive pain management integration utilizing holistic recovery approaches	Coordination with MAT schedules and pain management considerations
**CUD**	Moderate intensity protocols optimizing ECS activation for natural replacement	Standard protocols with enhanced mood regulation addressing motivational deficits	Limited evidence-based protocols requiring an individualized approach with cognitive monitoring	Enhanced mindfulness components supporting cognitive function and executive control	Standard safety protocols with mood monitoring and cognitive function assessment
**ADVANCED PROTOCOLS**					
**Low-Dose HIIT**	N/A	N/A	1 × 4 min interval ≥ 90% HRmax + 2 × 4 repetitions strength training ≥ 90% 1 RM	Gentle integration with HIIT recovery periods	Enhanced monitoring with immediate medical support
**High-Dose HIIT**	N/A	N/A	4 × 4 min intervals + 4 × 4 repetitions at ≥90% intensity, 3×/week for 8 weeks	Active recovery integration with mindfulness techniques	Continuous cardiac monitoring with emergency protocols
**Cognitive Enhancement Focus**	Standard aerobic protocols with cognitive assessment integration	Functional movement patterns supporting ADL performance	Structured intervals optimizing executive function recovery and processing speed	Attention training integration with movement-based meditation	Cognitive function monitoring with validated assessment tools
**QUALITY BENCHMARKS**					
**Process Measures**	Session attendance > 75% with systematic barrier identification and resolution	Program completion rates > 80% with dropout factor analysis and retention higher	Target intensity achievement > 85% sessions; strength progression tracking with cognitive monitoring	Session attendance > 86% with consistent practice development emphasis	Minor AE rates < 5% with zero serious events and incident analysis
**Clinical Outcomes**	Craving lower within 6–8 weeks with sustained abstinence > 60% at follow-up	Tx engagement > 80% with measurable self-efficacy improvements	Executive function improvement within 4–6 weeks; VO2max gains with strength increases	Stress lower with HPA axis normalization through physiological markers	Systematic incident reporting with continuous QI protocol implementation
**Long-term Sustainability**	Exercise habit maintenance at 3, 6, and 12 months assessments with community integration	Strength maintenance with continued progression and long-term fitness goal development	VO2max and strength improvements maintained at follow-up; sustained cognitive benefits	Lifestyle integration with stress management skill generalization beyond Tx	QI protocols with outcome monitoring and evidence-based program refinement
**HEALTHCARE INTEGRATION**					
**Staffing Requirements**	Exercise physiologist supervision with medical oversight, ensuring safety and therapeutic effectiveness	Qualified fitness professional with PT consultation for injury prevention	Certified HIIT specialist with cardiac rehabilitation training and emergency response certification	Certified instructor with MH integration supporting comprehensive recovery approaches	Emergency medical protocols with multidisciplinary coordination across specialties
**Facility Specifications**	Cardiorespiratory equipment with physiological monitoring devices ensuring safe exercise delivery	Muscle performance training equipment with functional movement space accommodating diverse modalities	Specialized HIIT equipment with advanced cardiac monitoring and immediate emergency access	Quiet, adaptable space with minimal distractions supporting meditative practices	Emergency equipment accessibility with medical support coordination, ensuring rapid response
**Documentation Standards**	Comprehensive session logs with physiological responses and individualized progress tracking	Strength assessments with progression records and functional improvement documentation	HIIT performance logs with heart rate data, recovery metrics, and cognitive assessment integration	Mindfulness practice logs with stress measure tracking supporting therapeutic progress	Medical clearance documentation with incident reports and outcome monitoring systems

Footer: Symbols Used: higher = increase/increased/improvement; lower = decrease/decreased/reduction; → = progression/advanced to. Clinical Abbreviations: AE = Adverse Event; AUD = Alcohol Use Disorder; BP = Blood Pressure; CUD = Cannabis Use Disorder; CV = Cardiovascular; ECG = Electrocardiogram; ECS = Endocannabinoid System; FITT = Frequency, Intensity, Time, Type; HPA = Hypothalamic–Pituitary–Adrenal; HR = Heart Rate; HRmax = Heart Rate Maximum; HRR = Heart Rate Reserve; hx = history; MAT = Medication-Assisted Treatment; MET = Metabolic Equivalent of Task; meds = medications; MH = Mental Health; MSK = Musculoskeletal; OUD = Opioid Use Disorder; PT = Physical Therapy; QI = Quality Improvement; ROM = Range of Motion; Tx = Treatment; 1 RM = One Repetition Maximum. FITT Model Definition: Frequency = How often exercise sessions occur (sessions per week); Intensity = How hard the exercise feels (percentage of maximum effort or heart rate); Time = Duration of each exercise session (minutes per session); Type = Specific exercise modality or activity selection (aerobics, resistance, mind–body). Quality Benchmark Definitions: Process Measures = Operational indicators of program delivery and participant engagement that can be tracked throughout intervention implementation; Clinical Outcomes = Direct therapeutic benefits related to substance use disorder recovery and treatment effectiveness measured through validated assessment tools; Long-term Sustainability = Maintenance of therapeutic benefits and continued exercise participation beyond formal treatment completion.

## Data Availability

Data sharing is not applicable to this article as no datasets were generated or analyzed during the current study.

## Supplementary Materials

The following supporting information can be downloaded at https://www.mdpi.com/article/10.3390/diseases14080272/s1, Table S1: Preferred Reporting Items for Systematic Reviews and Meta-Analyses extension for Scoping Reviews (PRISMA-ScR) Checklist [73,74,75].

## Abbreviations

The following abbreviations are used in this manuscript:

1 RM	One-repetition maximum
ACTH	Adrenocorticotropic hormone
AUD	Alcohol use disorder
BDNF	Brain-derived neurotrophic factor
CUD	Cannabis use disorder
CV	Cardiovascular
DSM-5	Diagnostic and Statistical Manual of Mental Disorders, Fifth Edition
ECG	Electrocardiogram
ECS	Endocannabinoid system
FITT	Frequency, intensity, time, type
FNDC5	Fibronectin type III domain-containing protein 5
HIIT	High-intensity interval training
HPA	Hypothalamic–pituitary–adrenal
HRmax	Heart rate maximum
HRR	Heart rate reserve
HRV	Heart rate variability
ICD-11	International Classification of Diseases, 11th Revision
IL-6	Interleukin-6
MAT	Medication-assisted treatment
MET	Metabolic equivalent of task
OUD	Opioid use disorder
PGC-1α	Peroxisome proliferator-activated receptor-gamma coactivator 1-alpha
PRISMA-ScR	Preferred Reporting Items for Systematic Reviews and Meta-analyses extension for Scoping Reviews
QoL	Quality of life
RCT	Randomized controlled trial
SMD	Standardized mean difference
SUCRA	Surface under the cumulative ranking curve
SUD	Substance use disorder
TNF-α	Tumor necrosis factor-alpha
