# Supplementary material for: Exercise Training Interventions as Therapeutic Approaches in Substance Use Disorder Treatment: A Scoping Review of Direct Clinical Outcomes and Implementation Strategies
Source: Diseases. 2026 Jul 27;14(8):272. doi: 10.3390/diseases14080272 (PMC13512151; doi:10.3390/diseases14080272)
Supplement: Supplementary file 1 [file diseases-14-00272-s001.zip › diseases-4347522-supplementary.pdf]

Supplementary Table S1. Preferred Reporting Items for Systematic Reviews and Meta-Analyses extension for Scoping Reviews (PRISMA-ScR) Checklist

SECTION	ITEM	PRISMA-ScR CHECKLIST ITEM	REPORTED ON PAGE #
**TITLE**			
Title	1	Identify the report as a scoping review.	p. 1 (title).
**ABSTRACT**			
Structured summary	2	Provide a structured summary that includes (as applicable): background, objectives, eligibility criteria, sources of evidence, charting methods, results, and conclusions that relate to the review questions and objectives.	p. 1 (Abstract).
**INTRODUCTION**			
Rationale	3	Describe the rationale for the review in the context of what is already known. Explain why the review questions/objectives lend themselves to a scoping review approach.	pp. 2-3 (Introduction; rationale for exercise interventions in SUD treatment and implementation gap).
Objectives	4	Provide an explicit statement of the questions and objectives being addressed with reference to their key elements (e.g., population or participants, concepts, and context) or other relevant key elements used to conceptualize the review questions and/or objectives.	p. 3 (Introduction; stated aims and PCC-aligned objectives).
**METHODS**			
Protocol and registration	5	Indicate whether a review protocol exists; state if and where it can be accessed (e.g., a Web address); and if available, provide registration information, including the registration number.	p. 3 (Methods 2.1; OSF registration doi.org/10.17605/OSF.IO/K3E8Z).
Eligibility criteria	6	Specify characteristics of the sources of evidence used as eligibility criteria (e.g., years considered, language, and publication status), and provide a rationale.	pp. 3 and 5-6 (Methods 2.2 and 2.5; eligibility criteria and rationale for exclusions).
Information sources*	7	Describe all information sources in the search (e.g., databases with dates of coverage and contact with authors to identify additional sources), as well as the date the most recent search was executed.	p. 4 (Methods 2.3; PubMed, Scopus, PsycINFO, SPORTDiscus; January 2000 to 31 December 2024).
Search	8	Present the full electronic search strategy for at least 1 database,	p. 4 (Methods 2.3; search domains, keywords, limits, and date range described).

St. Michael's
Inspired Care.
Inspiring Science.

SECTION	ITEM	PRISMA-ScR CHECKLIST ITEM	REPORTED ON PAGE #
		including any limits used, such that it could be repeated.	
Selection of sources of evidence†	9	State the process for selecting sources of evidence (i.e., screening and eligibility) included in the scoping review.	p. 4 (Methods 2.4; independent screening, calibration agreement, kappa, discrepancy resolution).
Data charting process‡	10	Describe the methods of charting data from the included sources of evidence (e.g., calibrated forms or forms that have been tested by the team before their use, and whether data charting was done independently or in duplicate) and any processes for obtaining and confirming data from investigators.	p. 6 (Methods 2.6; standardized data extraction/data charting form and duplicate extraction).
Data items	11	List and define all variables for which data were sought and any assumptions and simplifications made.	pp. 3-6 (Methods 2.2, 2.5, and 2.6; population, concept, context, interventions, comparisons, outcomes).
Critical appraisal of individual sources of evidence§	12	If done, provide a rationale for conducting a critical appraisal of included sources of evidence; describe the methods used and how this information was used in any data synthesis (if appropriate).	p. 6 (Methods 2.7; formal quality assessment not applied; methodological characteristics documented).
Synthesis of results	13	Describe the methods of handling and summarizing the data that were charted.	p. 6 (Methods 2.7; narrative synthesis framework).
**RESULTS**			
Selection of sources of evidence	14	Give numbers of sources of evidence screened, assessed for eligibility, and included in the review, with reasons for exclusions at each stage, ideally using a flow diagram.	p. 5 (Figure 1; PRISMA-ScR flow diagram with identification, screening, eligibility, and inclusion counts).
Characteristics of sources of evidence	15	For each source of evidence, present characteristics for which data were charted and provide the citations.	pp. 6-17 (Results sections 3-7 and Table 1).
Critical appraisal within sources of evidence	16	If done, present data on critical appraisal of included sources of evidence (see item 12).	p. 6 (Methods 2.7; not applicable because no formal critical appraisal was conducted for this scoping review).
Results of individual sources of evidence	17	For each included source of evidence, present the relevant data that were charted that relate to the review questions and objectives.	pp. 6-17 (Results sections 3-7 and Table 1; relevant charted data by modality, substance type, and outcomes).
Synthesis of results	18	Summarize and/or present the charting results as they relate to	pp. 6-17 and 23-27 (results synthesis and Discussion 10.1-10.4).

SECTION	ITEM	PRISMA-ScR CHECKLIST ITEM	REPORTED ON PAGE #
		the review questions and objectives.	
**DISCUSSION**			
Summary of evidence	19	Summarize the main results (including an overview of concepts, themes, and types of evidence available), link to the review questions and objectives, and consider the relevance to key groups.	pp. 23-27 (Discussion 10.1-10.4; summary of evidence, mechanisms, implementation contributions, and future directions).
Limitations	20	Discuss the limitations of the scoping review process.	pp. 26-27 (Discussion 10.4; study heterogeneity and methodological limitations).
Conclusions	21	Provide a general interpretation of the results with respect to the review questions and objectives, as well as potential implications and/or next steps.	pp. 27-28 (Conclusion; implications and next steps).
**FUNDING**			
Funding	22	Describe sources of funding for the included sources of evidence, as well as sources of funding for the scoping review. Describe the role of the funders of the scoping review.	p. 24 (Funding statement; no external funding).

JBI = Joanna Briggs Institute; PRISMA-ScR = Preferred Reporting Items for Systematic Reviews and Meta-Analyses extension for Scoping Reviews.
* Where *sources of evidence* (see second footnote) are compiled from, such as bibliographic databases, social media platforms, and Web sites.
† A more inclusive/heterogeneous term used to account for the different types of evidence or data sources (e.g., quantitative and/or qualitative research, expert opinion, and policy documents) that may be eligible in a scoping review as opposed to only studies. This is not to be confused with *information sources* (see first footnote).
‡ In scoping-review methodology, the process of extracting data is commonly referred to as data charting; this terminology is used in established scoping-review frameworks and JBI guidance. Full references are provided below.
§ The process of systematically examining research evidence to assess its validity, results, and relevance before using it to inform a decision. This term is used for items 12 and 19 instead of "risk of bias" (which is more applicable to systematic reviews of interventions) to include and acknowledge the various sources of evidence that may be used in a scoping review (e.g., quantitative and/or qualitative research, expert opinion, and policy document).

References

Approved PRISMA-ScR and methodology references formatted for inclusion at the end of the manuscript reference list. References are numbered 73-75 to follow the manuscript reference list, which ends at reference 72.

73. Rethlefsen, M.L.; Kirtley, S.; Waffenschmidt, S.; Ayala, A.P.; Moher, D.; Page, M.J.; Koffel, J.B.; PRISMA-S Group. PRISMA-S: An extension to the PRISMA Statement for Reporting Literature Searches in Systematic Reviews. Syst. Rev. 2021, 10, 39. https://doi.org/10.1186/s13643-020-01542-z.
74. Arksey, H.; O'Malley, L. Scoping studies: Towards a methodological framework. Int. J. Soc. Res. Methodol. 2005, 8, 19-32. https://doi.org/10.1080/1364557032000119616.
75. Levac, D.; Colquhoun, H.; O'Brien, K.K. Scoping studies: Advancing the methodology. Implement. Sci. 2010, 5, 69. https://doi.org/10.1186/1748-5908-5-69.
